# Diversity and abundance of ring nucleases in type III CRISPR-Cas loci

**DOI:** 10.1098/rstb.2024.0084

**Published:** 2025-09-04

**Authors:** Ville Hoikkala, Haotian Chi, Sabine Grüschow, Shirley Graham, Malcolm F White

**Affiliations:** ^1^ School of Biology, University of St Andrews, St Andrews, UK

**Keywords:** CRISPR, phosphodiesterase, ring nuclease, cyclic nucleotide

## Abstract

Most type III CRISPR-Cas systems facilitate immune responses against invading mobile genetic elements such as phages by generating cyclic oligoadenylates (cOAs). Downstream effectors activated by cOAs are typically non-specific proteins that induce damage to essential cellular components, thereby preventing phage epidemics. Owing to these toxic effects, it is crucial that the production and concentration of cOAs remain under tight regulatory control during infection-free periods or when deactivating the immune response after clearing an infection. Type III CRISPR loci often encode enzymes known as ring nucleases (RNs) that bind and degrade specific cOAs, while some effectors are auto-deactivating. Despite the discovery of several classes of RNs, a comprehensive bioinformatic analysis of type III CRISPR-Cas loci in this context is lacking. Here, we examined 38 742 prokaryotic genomes to provide a global overview of type III CRISPR loci, focusing on the known and predicted RNs. The candidate RNs Csx16 and Csx20 are confirmed as active enzymes, joining Crn1–3. Distributions and patterns of co-occurrence of RNs and associated effectors are explored, allowing the conclusion that a sizeable majority of type III CRISPR systems regulate cOA levels by degrading the signalling molecules, which has implications for cell fate following viral infection.

This article is part of the discussion meeting issue ‘The ecology and evolution of bacterial immune systems’.

## 1. Introduction

CRISPR-Cas is an adaptive prokaryotic immune system that incorporates genomic fragments of invading mobile genetic elements (MGEs) into the host’s chromosomal CRISPR array [1,2]. These fragments, called spacers, are expressed during subsequent infections as CRISPR RNA (crRNA) and help interference complexes to target invading nucleic acids via sequence complementarity [3]. Type III CRISPR-Cas systems use multi-subunit interference complexes, hallmarked by Cas10—a large protein that can harbour two main functions [3]. While a minority of Cas10 proteins use an HD (histidine-aspartate) nuclease domain to cleave single stranded DNA non-specifically [4,5], over 90% of Cas10s have a cyclase domain that generates second messenger signalling molecules [6–9]. The signal molecules produced by Cas10, either cyclic oligoadenylates (cOA) or S-adenosyl methionine-AMP [10], accumulate in the cell and activate downstream effector proteins that are typically encoded by genes in the same operon. The effector proteins contain a sensory domain that captures the signal, leading to the allosteric activation of the effector domain that may be a nuclease, protease or a membrane-disrupting domain (see reviews [11,12]). Activation of effectors thus tends to have toxic consequences for cells and their actions can lead to growth arrest. This outcome is sometimes called abortive infection; an umbrella term under which diverse immune mechanisms are often grouped, all sharing the ecological rationale of sacrificing the host for the sake of the population (see review [13]). Whether phage defence mechanisms actually lead to cell death *in vivo* is debated [14], and the presence of ring nucleases (RNs) in CRISPR type III systems provides a mechanism to avoid this outcome.

RNs are small proteins that degrade cOA signal molecules, thus thwarting the activation of downstream effectors. They are generally encoded in CRISPR-Cas loci and are also used by viruses as anti-CRISPR (Acr) proteins [15]. It is not currently known whether their primary function in cells is to deactivate defence in an ongoing infection state or to curtail signal molecule levels in a non-infected state. To date, three families of RNs have been experimentally verified. The first RN to be discovered, CRISPR-associated ring nuclease 1 (Crn1), was identified biochemically from a lysate of *Saccharolobus solfataricus* [16]. Crn1 is a metal-independent phosphodiesterase that binds cA_4_ in its dimeric CRISPR-associated Rossmann fold (CARF) domains and degrades it into linear A_2_ > P (cyclic 2′,3′ phosphate) products. Crn1 is most commonly found in crenarchaeal type III CRISPR loci and has a relatively low cA_4_ cleavage rate, which may be tuned to remove cA_4_ from the cell without disrupting the immune response [16].

Crn2 and its virally encoded counterpart AcrIII-1 are also cA_4_-cleaving RNs [15]. This class of RN is characterized by the DUF1874 domain, which forms a dimeric cA_4_ recognition domain. AcrIII-1 degrades cA_4_ much more quickly than Crn1, consistent with its role in subverting type III CRISPR signalling during viral infection. This family of RNs has also been found fused to the bacterial effector Csx1, constituting a self-limiting cA_4_-activated ribonuclease [17].

Crn3, previously known as Csx3, constitutes the third family of cA_4_-cleaving RNs. The structure of Crn3 is only distantly related to the CARF superfamily, harbouring closer resemblance to sulfate transporter and anti-sigma factor antagonist domains [18–20]. Crn3 binds cA_4_ by sandwiching the molecule between two adjacent dimers that tetramerize in a head-to-tail orientation [18]. The phosphodiesterase reaction is manganese dependent and generates linear A_2_-P products. Crn3 is sometimes fused to an AAA-ATPase domain of unknown function [20,21].

In addition to the dedicated RNs, several type III CRISPR effectors have been shown to degrade their cOA activators within the binding site of the sensory domain, effectively functioning as self-limiting enzymes. All cA_6_-dependent Csm6 family ribonucleases studied to date display cA_6_ RN activity in the CARF recognition domain [22–24]. Likewise cA_4_-activated Csm6/Csx1 effectors are also capable of degrading their activator within the CARF domain [25–27]. Recently, the CalpL effector, which uses a SAVED (SMODS-associated and fused to various effector domains) domain to bind cA_4_, has also been confirmed as an RN [28,29].

The CRISPR-associated Csx15, Csx16 and Csx20 proteins have been predicted to be RNs, based on sequence and genomic neighbourhood analyses [20], but this has not yet been experimentally confirmed. RNs are thus common in type III CRISPR systems but have not been studied systematically. Here, we undertook an extensive analysis of type III CRISPR loci, mapping the known and predicted RNs and exploring their association with the diverse range of effector proteins. The cA_4_ RN activities of Csx15, Csx16 and Csx20 are investigated biochemically, and structural modelling is used to predict their mechanisms of cA_4_ recognition.

## 2. Material and methods

### (a) Data preparation

A total of 38 742 complete bacterial and archaeal genomes were downloaded from NCBI on 25 March 2024. For phage genomes, the May 2024 set of 28 114 curated genomes from the Millard database [30] was downloaded.

### (b) Bioinformatic methods

A previously described, the Snakemake [31] pipeline was used as the basis for type III CRISPR locus characterization [6]. In short, the pipeline uses custom-built Cas10 Hidden Markov Model (HMM) profiles to find type III CRISPR loci, which are further analysed using CCTyper [32] and a panel of HMM profiles to discover effector proteins. The pipeline was modified to enable ring nuclease detection and phage genome analysis. Custom-built HMM-libraries were constructed using published sequence data in NCBI for ring nucleases Crn1, Crn2 and Crn3. For Csx15, Csx16 and Csx20, libraries were built based on blastp homology searches against the NCBI protein database using previously published sequences [20] as queries. The predicted RN Csx14 [20] appears to be a member of the Crn1 family and was included in the HMM profiles for Crn1. Each protein coding sequence within 6 kbp of a type III CRISPR locus was analysed for RNs using hmmscan from the Hmmer 3.3.2 package [33]. To prevent cross-annotation with effectors that have similar domains (e.g. CARF), there was a maximum length cut-off of 250 amino acids for RNs during annotation. Candidate RNs were screened by multiple sequence alignment and manual inspection to check for the presence of absolutely conserved residues. A small number of false positives, lacking key highly conserved residues, were identified and removed. Crn1 was noted previously as a member of the CARF7 and CARF_m13 families [20]. Attempts to assign the other RNs into CARF families using phylogenetic and clustering approaches was unsuccessful owing to high sequence/structural divergence. A table of all cellular and phage-encoded RNs including annotation details and sequences is provided in the electronic supplementary material, S1.

The steps for constructing the Cas10 phylogenetic tree were outlined in [6]. In short, each Cas10 was annotated for the presence of cyclase or nuclease domains. The Cas10 sequences were then aligned with Muscle [34]. A phylogenetic tree file was built using FastTree2 [35] with arguments -wag and -gamma, and visualized in RStudio 2024.4.0.735 [36] using ggtree [37]. The tree was annotated with CRISPR subtype data from CCTyper (in a few cases corrected manually) and with the presence of known and candidate ring nuclease families. The signal molecule associated with each locus was inferred from the type(s) of effector present and annotated for each locus in the tree.

A network interaction graph was made with Gephi (https://gephi.org/) using RN/effector co-occurrence data. Co-occurrences between effectors and between RNs were removed to highlight those between RNs and effectors.

To search for ring nucleases in phage genomes, the proteomes of all 28 114 phage genomes in the Millard phage database were analysed using our RN HMM libraries using Hmmer 3.3.2 [33] similar to the type III CRISPR-Cas analysis outlined above. Hits were analysed manually using multiple sequence alignment to detected conserved residues, and a few false-positive hits were removed manually. CARF families based on motifs were assigned as with the CRISPR RNs. The phage-encoded RNs with annotation details and phage data are provided in the electronic supplementary material, S1.

### (c) Cloning, expression and purification of Csx15, 16 and 20

Synthetic genes (electronic supplementary material, table S1) encoding Csx15, Csx16 and Csx20, codon optimized for expression in *Escherichia coli*, were purchased from Integrated DNA Technologies (IDT), Coralville, USA, cloned into the pEHisV5TEV vector [38] between the *Nco*I and *Bam*HI sites and transformed into DH5α cells. Construct integrity was confirmed by sequencing (Eurofins Genomics, DE).

The constructs were transformed into *E. coli* C43 (DE3) and proteins were expressed according to the standard protocols previously described [38]. In brief, 2 l of culture were induced with 0.4 mM isopropyl-β-D-1-thiogalactoside at an optical density of approximately 0.8 and grown for 4 h or overnight at 25°C. Cells were harvested (4000 rpm; Beckman Coulter JLA-8.1 rotor) and resuspended in lysis buffer and lysed by sonication. Proteins were purified with an immobilized metal affinity chromatography (IMAC) column (HisTrapFF, Cytiva, Marlborough, USA), washed with five column volumes of loading buffer and eluted with a linear gradient of loading buffer plus 0.5 M imidazole. Following his-tag removal by tobacco etch virus (TEV) protease, proteins were subjected to a second IMAC step and the unbound fraction collected. Size exclusion chromatography was used to further purify the proteins, which were eluted isocratically as described previously [38]. Pure proteins were concentrated, aliquoted and stored frozen at −70°C.

### (d) Ring nuclease activity of Csx15, Csx16 and Csx20

Ring nuclease activity was assayed by incubating 1 µM of each protein with 100 µM synthetic cA_3_, cA_4_ or cA_6_ (Biolog) in reaction buffer (20 mM Tris-HCl pH7.5, 250 mM NaCl and 5 mM ethylenediaminetetraacetic acid (EDTA) at 30°C for 60 min. The reaction was quenched by adding methanol and vortexing. The mixture was then dried, before resuspension in water for high pressure liquid chromatography (HPLC) analysis on an UltiMate3000 HPLC system (Thermo Fisher scientific) with a C18 column (Kinetex EVO 2.1 × 50 mm, particle size 2.6 μm). The column temperature was set at 40°C and absorbance was monitored at 260 nm. Samples were analysed by gradient elution with solvent A (20 mM ammonium acetate, pH 8.5) and solvent B (methanol) as a flow rate of 0.3 ml min^−1^ as follows: 0−0.5 min, 1% B; 0.5−6 min, 1–15% B; 6−7 min, 100% B.

## 3. Results

### (a) Structural models and ring nuclease activity of Csx15, Csx16 and Csx20

We showed previously that 92% of type III CRISPR loci probably function via nucleotide signalling, with active Cas10 polymerase domains [6]. Of these, the predominant signalling molecule is cA_4_, so it is perhaps unsurprising that the three stand-alone RNs identified to date, Crn1−3, are all specific for cA_4_ [15,16,18]. Csx15, 16 and 20 were previously identified as candidate RNs based on genome context, sequence conservation and structural predictions [20]. However, these predictions have not been confirmed biochemically, so we first sought to test this specific hypothesis.

**Figure 1.**
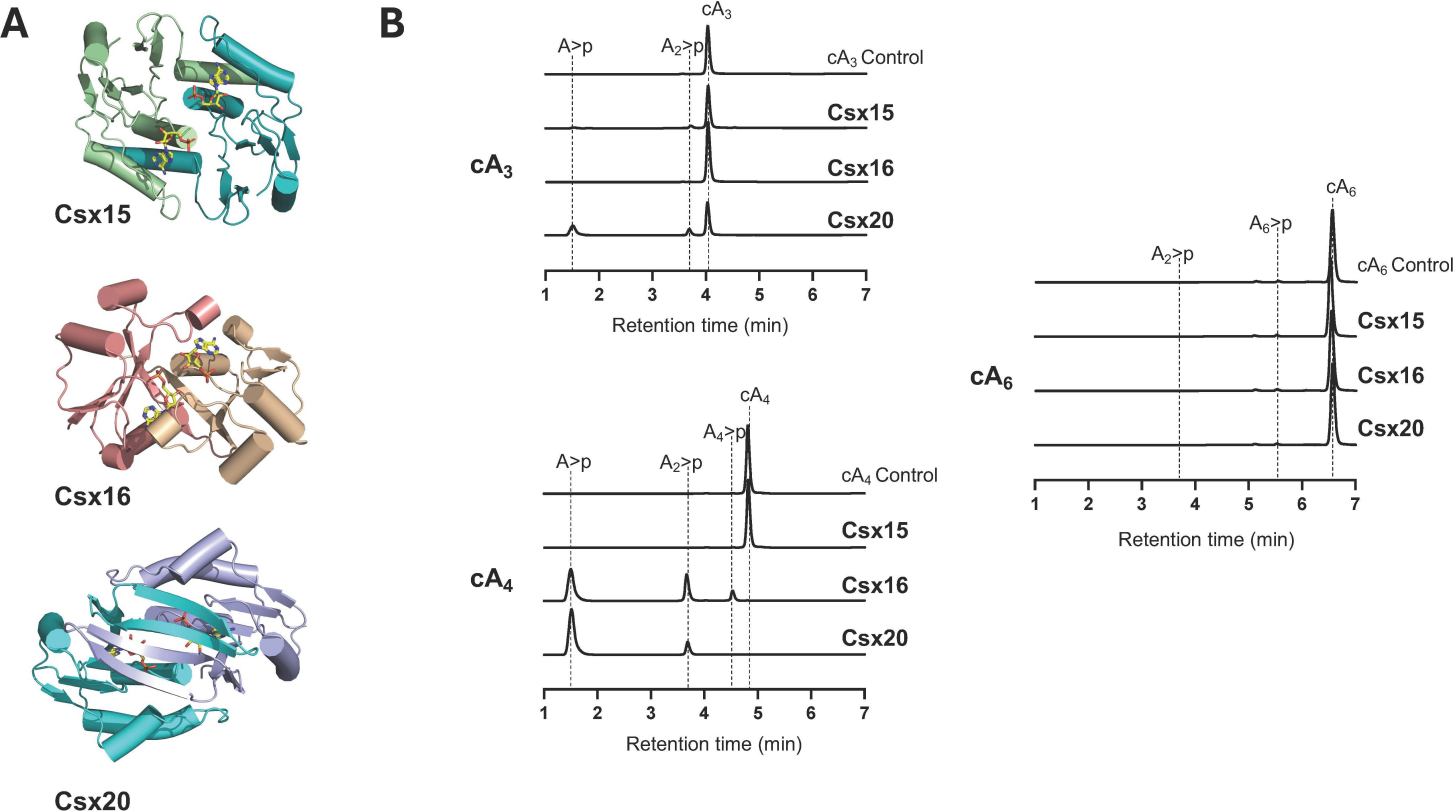
Structural modelling and RN activity of Csx15, Csx16 and Csx20. (A) Dimeric protein AF3 models are shown with 2 AMP molecules (yellow sticks) modelled to mimic the cOA binding site, subunits are coloured differently for ease of interpretation. (B) RN activity of Csx15, Csx16 and Csx20 against cA_3_, cA_4_ and cA_6_, monitored by HPLC. Csx16 and Csx20 degrade cA_4_ into linear products. Standards and characterized reaction products are labelled. (>*p* represents a 2′,3′-cyclic phosphate).

The structures of Csx15, Csx16 and Csx20 were modelled as dimers using Alphafold3 (AF3) ([39]; figure 1A). Two AMP ligands were included to help predict cOA binding sites. Inclusion of these ligands improved the ordering of the mobile loops that probably become structured on cOA binding. All three models were predicted with high confidence (ptm/iptm/ranking scores 0.91/0.90/0.94, 0.79/0.75/0.77 and 0.95/0.94/0.95 for Csx15, 16 and 20, respectively). To gain more insight into the likely cOA binding sites of the proteins, we mapped conserved residues from a diverse range of homologues for each protein (electronic supplementary material, figure S1) onto the AF3 structures (electronic supplementary material, figure S2). This revealed a cluster of conserved residues on one face of Csx16 and Csx20 that correspond with the modelled AMP binding sites and probably pinpoint the binding site for cOA. However, for Csx15, conserved residues were more prevalent on the ‘bottom’ face of the dimer, away from the canonical cOA binding site. This situation is reminiscent of Crn3, which forms filaments of head-to-tail dimers sandwiching cA_4_ [18]. Structural comparisons using the DALI server [40] yielded hits for Csx15 with SAVED and CARF family proteins (*Z*-score > 5 for PDB accessions 7RWM, 8Q3Z, 8FMF and 7QDA); Csx16 yielded a significant hit (*Z*-score 4.8) with PDB 2J6B—a viral homologue of Crn2 [41] while Csx20 yielded a strong match (*Z*-score 8.0) with the Crn1 family protein Sso2081 (PDB 7YGH) [42]. Pairwise comparison of the predicted structures of Csx16 and Csx20 yielded a DALI *Z*-score of 8.9, consistent with a common core fold consisting of a 5-stranded β-sheet flanked by α-helices. These structural features are consistent with a derived Rossmann fold (CARF-like) structure for Csx15, Csx16 and Csx20, an observation reinforced by the two-dimensional topology maps [43] of the three predicted proteins and comparison with Crn1 (electronic supplementary material, figure S3).

Representative examples of Csx15, Csx16 and Csx20 were cloned, expressed in *E. coli* and purified to homogeneity as described in the methods (electronic supplementary material, figure S4A). The proteins were tested individually for the ability to degrade cA_3_, cA_4_ and cA_6_ under conditions of 100-fold substrate excess for 1 h (figure 1B). Csx16 and Csx20 showed clear RN activity against cA_4_, fully degrading it to small linear products. Neither enzyme degraded cA_6_, while only very minor activity for Csx20 against cA_3_ could be observed. Csx15, on the other hand, displayed no RN activity against any cOA species (figure 1B). To explore RN activity more fully, we repeated these experiments with a 10-fold higher concentration of protein (10 µM) (electronic supplementary material, figure S4B). Under these conditions, Csx20 fully degraded cA_3_ as well as cA_4_, while Csx16 retained specificity for cA_4_. Very limited activity of Csx15 against cA_3_ and cA_6_, but not cA_4_, could be observed. We conclude that Csx16 and Csx20 are cA_4_-specific RNs, while the function of Csx15 remains uncertain. It is possible that we have not found the correct reaction conditions to reveal the activity of Csx15. Alternatively, it could conceivably act as a cOA ‘sponge’ rather than a RN, as phage-encoded sponge proteins are known to sequester cyclic nucleotides and inhibit cellular defences [44,45].

### (b) Distribution and co-occurrence patterns of extrinsic ring nucleases

**Figure 2.**
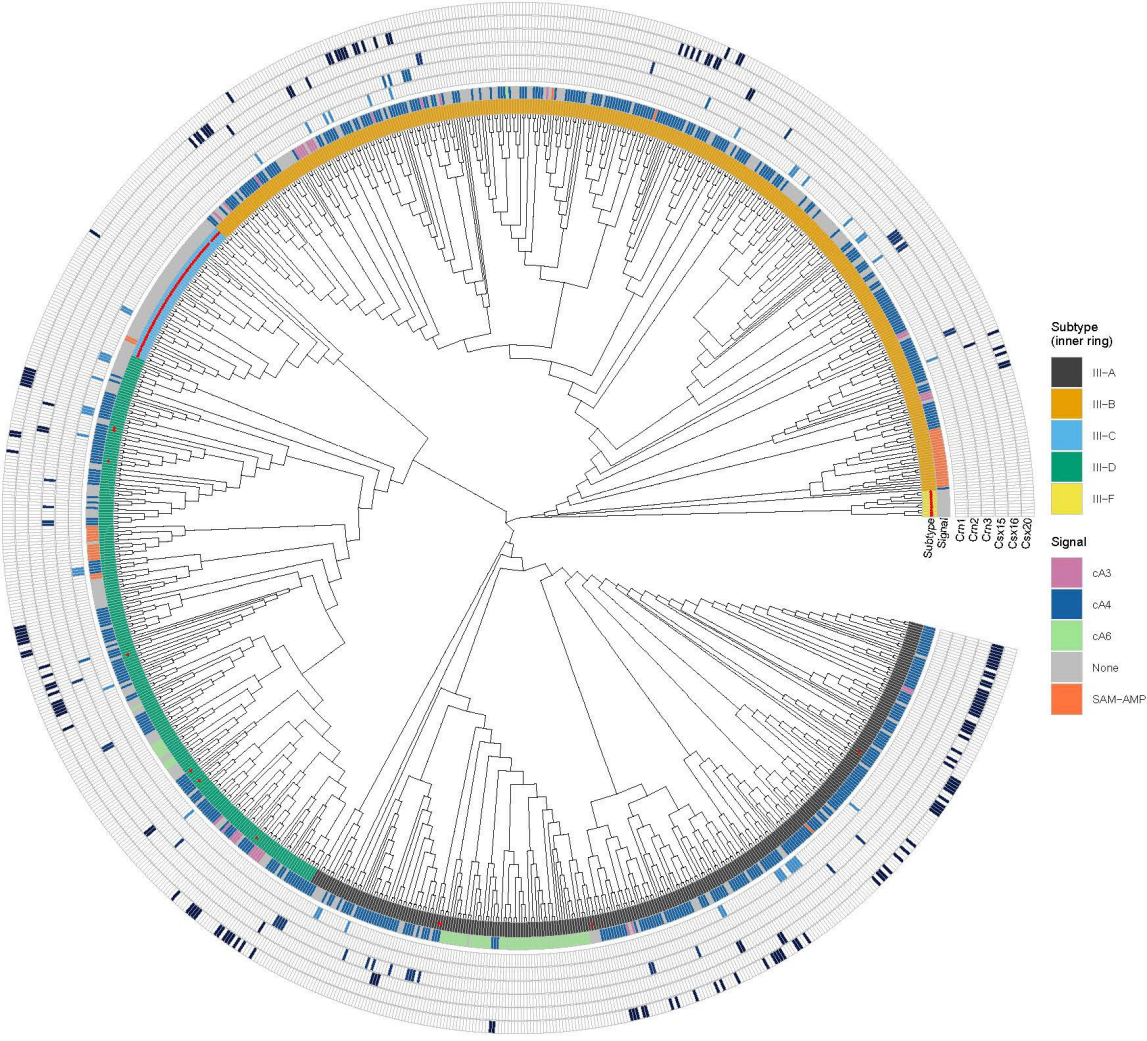
Phylogenetic tree of Cas10 with ring nuclease distribution. The signalling molecule used by each system is predicted based on effector content of the locus, as defined in [6], and coloured according to the key. Instances of candidate ring nucleases Crn1, Crn2, Crn3, Csx15, Csx16 and Csx20 are indicated in concentric circles. Cas10s lacking a clear active cyclase domain are indicated by a red dot in the subtype ring.

Having explored the RN activity of Csx15, 16 and 20, we proceeded to analyse the distribution of extrinsic RNs across the Cas10 tree (figure 2). This analysis immediately demonstrated that RNs are widespread in Cas10 loci, and when found in an effector-containing locus (96% of RN instances), they are associated only with cA_4_-dependent effectors. In the 536 CRISPR-Cas loci that contained a cA_4_-activated effector, 211 (39%) had an associated RN. In 31 cases, RNs were found in loci with no effector, suggesting either the presence of unknown effectors in the locus or a role for RNs *in trans* by another type III CRISPR locus with a known effector. The most frequently observed RN was Csx20 (78 loci), followed by Csx16 (68 loci), Crn1 (44 loci), Crn3 (37 loci), Csx15 (18 loci) and Crn2 (7 loci). There was no obvious bias in the distribution of RNs with respect to CRISPR subtypes that use cOA signalling, while loci from subtypes III-C and III-F, which lack an active Cas10 cyclase, have very few associated RNs. Csm6 and Csm6-2, which are activated by cA_6_, are not found in association with known RNs, which may reflect intrinsic RN activity by these effectors.

Only 10 of the 242 RN-positive loci (approx. 4%) contained more than one RN, which supports the hypothesis that they are all performing equivalent roles. An exception to this rule was Csx15, which co-occurs with Crn1 in seven loci and as the sole RN in 11 loci. We investigated the frequency of Csx15 across all approximately 40 000 RefSeq genomes and found that Csx15 is found in genomes without a type III CRISPR-Cas locus in 25% of cases. Along with the peculiar co-occurrence pattern with Crn1 and lack of RN activity *in vitro*, this suggests Csx15 may not be a canonical RN.

**Figure 3.**
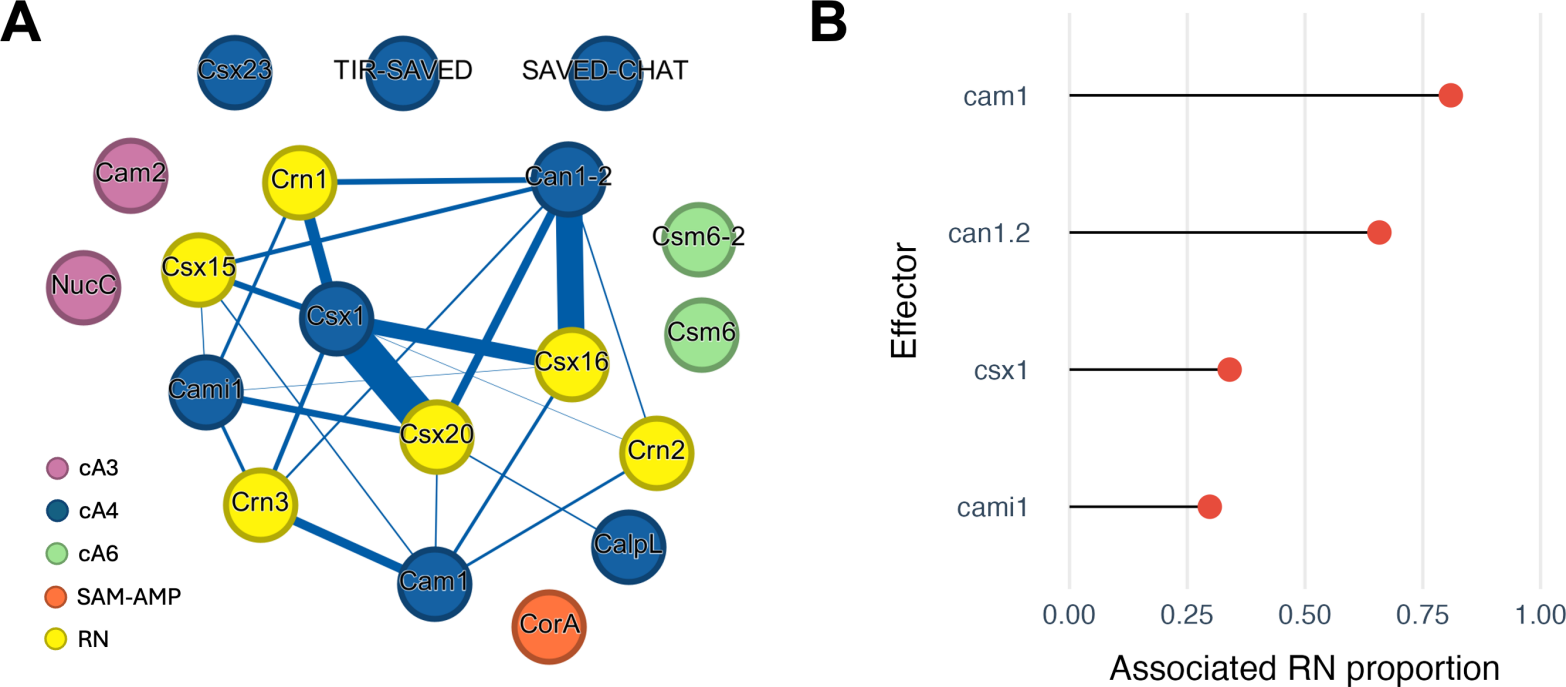
Co-occurrences of ring nucleases and effectors in type III CRISPR loci. (A) Gephi plot showing co-occurrence patterns between effectors and ring nucleases. Ring nucleases are in yellow, cA_3_, cA_4_ and cA_6_-activated effectors in purple, blue and green, respectively and SAM-AMP-activated effectors in orange. (B) The proportion of effector instances that are associated with a RN. The data only includes loci with one effector and zero or one RNs.

The co-occurrence of ring nucleases and effectors is shown in figure 3A. All characterized ring nucleases preferentially degrade cA_4_, so it is unsurprising (but reassuring) that they co-occur with cA_4_-specific effectors. The most abundant effector in our dataset, Csx1, defined here as the cA_4_-activated family of CARF-HEPN dimeric effector proteins [6] is found in association with each of the ring nucleases analysed here and has a particularly strong association with Csx20. We also analysed loci with a single known effector to quantify the proportion that encoded a RN (figure 3B). The observation that only 34% of Csx1-containing loci encode a known RN may be at least partially explained by the fact that some Csx1 family members have intrinsic RN activity [25,27]. In support of this, the Can1−2 effector family [46–48], which lack intrinsic RN activity, are associated with RNs in 66% of loci whereas for Cami1, which possesses RN activity [49,50], the proportion is only 30%. A similar pattern of association was identified by Makarova & Koonin [20]. Following this logic, the observation that the membrane bound effector Cam1 is found alongside RNs in 81% of loci suggests that this effector also lacks intrinsic RN activity, although that has yet to be tested [51]. None of the characterized cA_4_-specific RNs are found associated with effectors that use a different signalling molecule. While some of these effectors, such as Csm6, are effective RNs in their own right [22–24,27], others, such as NucC, are not [52]. This could suggest that RNs targeting molecules other than cA_4_ simply await discovery, or alternatively that infection outcomes are different in these cases.

Phage genomes were also investigated for ring nucleases. Among the 28 114 phage genomes in the Millard database, the previously described anti-CRISPR protein AcrIII-1 (the phage-encoded homologue of Crn2) [15] was by far the most abundant RN, present in 36 genomes. Additionally, Csx20 was found in four genomes and Csx16 in two genomes. The high relative abundance of AcrIII-1/Crn2 in phage genomes compared to its presence in only seven prokaryotic genomes supports the view that it probably originated from viruses as an anti-CRISPR and was subsequently adopted by cellular hosts to regulate cOA levels.

## 4. Discussion

By confirming the RN activity of Csx16 and 20, we have expanded the catalogue of extrinsic RNs to five families. These are widely distributed across the type III CRISPR systems that signal via cA_4_—the predominant signalling molecule used by 84% of all known effector instances [6]. When combined with the observation that intrinsic RN activity is frequently observed in CARF or SAVED sensor domains of effectors, we can conclude with some confidence that an ‘off-switch’ is an important component of most type III CRISPR systems. Exceptions, such as the prophage-encoded systems described in *Vibrio* species that use NucC or Csx23 effectors [52,53], exist but probably represent a small minority.

The prokaryotic immune system has two major components that potentiate an antiviral response by generation of cOA species: type III CRISPR-Cas and CBASS (cyclic oligonucleotide-based antiphage signalling system) [54]. Each system responds to infection by activating a specialized cyclase that generates a cyclic nucleotide second messenger which in turn activates one or more effector proteins to provide an immune response. CBASS and type III CRISPR-Cas have much in common, with shared signalling molecules such as cA_3_ and effectors such as NucC [52,55] and TIR-SAVED [6,56]. Each uses the signal amplification possible with a second messenger to activate a toxic response to infection that slows down viral replication at the cost of cell fitness. Their main point of divergence appears to be the fact that type III CRISPR systems often encode an ‘off-switch’ while, by contrast, CBASS activation appears to be a ‘one way street’ that may often result in cell death or growth arrest [57].

This fundamental difference between type III CRISPR and CBASS defence, despite all their similarities, may reflect the fact that the former can be activated early in infection (by viral mRNA) whilst the latter is viewed as a ‘last ditch’ defence, typically activated late in the infection cycle (for example, by intracellular phage capsid proteins [44]) when other defences have failed [58,59]. CBASS defence, functioning at the level of herd immunity, is clearly worth having, as the system is fairly common in bacteria [60]. Nevertheless, cells appear to avoid altruistic suicide when possible. One significant caveat is that RNs may primarily function to prevent aberrant activation of type III CRISPR-Cas defence (i.e. activation of Cas10 by non-viral RNA), rather than to clear up after a *bona fide* infection. The growth-arrested state facilitated by type III CRISPR-Cas may also enable other defence systems to clear the infecting virus, which could then be followed by reversal of the arrested state by RNs. Such cooperation between defence systems has been found between the RNA-targeting type VI CRISPR-Cas systems and restriction modification, where restriction enzymes cleave phage genome while the cell is under Cas13-induced dormancy [61]. Looking ahead, there is a pressing need for studies that tackle these questions using an evolutionary, population-based approach.

Although we have analysed six candidate RN families that account for a large proportion of the cA_4_-signalling CRISPR systems, it is possible and, indeed, likely that further RN families remain to be discovered. The candidate RN Unk_01 [20] appears unrelated to the CARF superfamily and was not examined here, but is worthy of further study. The function of Csx15 also remains an open question at this point. By looking closely at the loci which lack a known RN and which have an effector with intrinsic RN activity, we hope to unveil new RN families in the future.

## Data Availability

The Snakemake pipeline and accompanying scripts are available for review at [62]. This repository also contains the HMM profiles for Cas10s, effectors and all six ring nuclease families in this study. An interactive website for browsing the bioinformatic results is available at https://vihoikka.github.io/rn_browser. Supplementary material is available online [63].

## References

[1] Barrangou R, Fremaux C, Deveau H, Richards M, Boyaval P, Moineau S, Romero DA, Horvath P. 2007 CRISPR provides acquired resistance against viruses in prokaryotes. Science **315**, 1709–1712. (10.1126/science.1138140) 17379808

[2] Mojica FJM, Díez-Villaseñor C, García-Martínez J, Soria E. 2005 Intervening sequences of regularly spaced prokaryotic repeats derive from foreign genetic elements. J. Mol. Evol. **60**, 174–182. (10.1007/s00239-004-0046-3) 15791728

[3] Makarova KS *et al*. 2015 An updated evolutionary classification of CRISPR–Cas systems. Nat. Rev. Microbiol. **13**, 722–736. (10.1038/nrmicro3569) 26411297 PMC5426118

[4] Jung TY, An Y, Park KH, Lee MH, Oh BH, Woo E. 2015 Crystal structure of the Csm1 subunit of the Csm complex and its single-stranded DNA-specific nuclease activity. Structure **23**, 782–790. (10.1016/j.str.2015.01.021) 25773141

[5] Samai P, Pyenson N, Jiang W, Goldberg GW, Hatoum-Aslan A, Marraffini LA. 2015 Co-transcriptional DNA and RNA cleavage during type III CRISPR-Cas immunity. Cell **161**, 1164–1174. (10.1016/j.cell.2015.04.027) 25959775 PMC4594840

[6] Hoikkala V, Graham S, White MF. 2024 Bioinformatic analysis of type III CRISPR systems reveals key properties and new effector families. Nucleic Acids Res. **52**, 7129–7141. (10.1093/nar/gkae462) 38808661 PMC11229360

[7] Kazlauskiene M, Kostiuk G, Venclovas Č, Tamulaitis G, Siksnys V. 2017 A cyclic oligonucleotide signaling pathway in type III CRISPR-Cas systems. Science **357**, 605–609. (10.1126/science.aao0100) 28663439

[8] Niewoehner O, Garcia-Doval C, Rostøl JT, Berk C, Schwede F, Bigler L, Hall J, Marraffini LA, Jinek M. 2017 Type III CRISPR–Cas systems produce cyclic oligoadenylate second messengers. Nature **548**, 543–548. (10.1038/nature23467) 28722012

[9] Wiegand T, Wilkinson R, Santiago-Frangos A, Lynes M, Hatzenpichler R, Wiedenheft B. 2023 Functional and phylogenetic diversity of Cas10 proteins. CRISPR J. **6**, 152–162. (10.1089/crispr.2022.0085) 36912817 PMC10123807

[10] Chi H, Hoikkala V, Grüschow S, Graham S, Shirran S, White MF. 2023 Antiviral type III CRISPR signalling via conjugation of ATP and SAM. Nature **622**, 826–833. (10.1038/s41586-023-06620-5) 37853119 PMC10600005

[11] Athukoralage JS, White MF. 2022 Cyclic nucleotide signaling in phage defense and counter-defense. Annu. Rev. Virol. **9**, 451–468. (10.1146/annurev-virology-100120-010228) 35567297

[12] Steens JA, Salazar CRP, Staals RHJ. 2022 The diverse arsenal of type III CRISPR–Cas-associated CARF and SAVED effectors. Biochem. Soc. Trans. **50**, 1353–1364. (10.1042/bst20220289) 36282000 PMC9704534

[13] Lopatina A, Tal N, Sorek R. 2020 Abortive infection: bacterial suicide as an antiviral immune strategy. Annu. Rev. Virol. **7**, 1–14. (10.1146/annurev-virology-011620-040628) 32559405

[14] Fernández-García L, Wood TK. 2023 Phage-defense systems are unlikely to cause cell suicide. Viruses **15**, 1795. (10.3390/v15091795) 37766202 PMC10535081

[15] Athukoralage JS, McMahon SA, Zhang C, Grüschow S, Graham S, Krupovic M, Whitaker RJ, Gloster TM, White MF. 2020 An anti-CRISPR viral ring nuclease subverts type III CRISPR immunity. Nature **577**, 572–575. (10.1038/s41586-019-1909-5) 31942067 PMC6986909

[16] Athukoralage JS, Rouillon C, Graham S, Grüschow S, White MF. 2018 Ring nucleases deactivate type III CRISPR ribonucleases by degrading cyclic oligoadenylate. Nature **562**, 277–280. (10.1038/s41586-018-0557-5) 30232454 PMC6219705

[17] Samolygo A, Athukoralage JS, Graham S, White MF. 2020 Fuse to defuse: a self-limiting ribonuclease-ring nuclease fusion for type III CRISPR defence. Nucleic Acids Res. **48**, 6149–6156. (10.1093/nar/gkaa298) 32347937 PMC7293037

[18] Athukoralage JS, McQuarrie S, Grüschow S, Graham S, Gloster TM, White MF. 2020 Tetramerisation of the CRISPR ring nuclease Crn3/Csx3 facilitates cyclic oligoadenylate cleavage. eLife **9**, e57627. (10.7554/elife.57627) 32597755 PMC7371418

[19] Brown S, Gauvin CC, Charbonneau AA, Burman N, Lawrence CM. 2020 Csx3 is a cyclic oligonucleotide phosphodiesterase associated with type III CRISPR–Cas that degrades the second messenger cA4. J. Biol. Chem. **295**, 14963–14972. (10.1074/jbc.ra120.014099) 32826317 PMC7606696

[20] Makarova KS, Timinskas A, Wolf YI, Gussow AB, Siksnys V, Venclovas Č, Koonin EV. 2020 Evolutionary and functional classification of the CARF domain superfamily, key sensors in prokaryotic antivirus defense. Nucleic Acids Res. **48**, 8828–8847. (10.1093/nar/gkaa635) 32735657 PMC7498327

[21] Shah SA, Alkhnbashi OS, Behler J, Han W, She Q, Hess WR, Garrett RA, Backofen R. 2019 Comprehensive search for accessory proteins encoded with archaeal and bacterial type III CRISPR- cas gene cassettes reveals 39 new cas gene families. RNA Biol. **16**, 530–542. (10.1080/15476286.2018.1483685) 29911924 PMC6546367

[22] Garcia-Doval C, Schwede F, Berk C, Rostøl JT, Niewoehner O, Tejero O, Hall J, Marraffini LA, Jinek M. 2020 Activation and self-inactivation mechanisms of the cyclic oligoadenylate-dependent CRISPR ribonuclease Csm6. Nat. Commun. **11**, 1596. (10.1038/s41467-020-15334-5) 32221291 PMC7101355

[23] McQuarrie S, Athukoralage JS, McMahon SA, Graham S, Ackermann K, Bode BE, White MF, Gloster TM. 2023 Activation of Csm6 ribonuclease by cyclic nucleotide binding: in an emergency, twist to open. Nucleic Acids Res. **51**, 10590–10605. (10.1093/nar/gkad739) 37747760 PMC10702470

[24] Smalakyte D, Kazlauskiene M, F. Havelund J, Rukšėnaitė A, Rimaite A, Tamulaitiene G, Færgeman NJ, Tamulaitis G, Siksnys V. 2020 Type III-A CRISPR-associated protein Csm6 degrades cyclic hexa-adenylate activator using both CARF and HEPN domains. Nucleic Acids Res. **48**, 9204–9217. (10.1093/nar/gkaa634) 32766806 PMC7498309

[25] Athukoralage JS, Graham S, Grüschow S, Rouillon C, White MF. 2019 A type III CRISPR ancillary ribonuclease degrades its cyclic oligoadenylate activator. J. Mol. Biol. **431**, 2894–2899. (10.1016/j.jmb.2019.04.041) 31071326 PMC6599890

[26] Du L, Zhu Q, Lin Z. 2024 Molecular mechanism of allosteric activation of the CRISPR ribonuclease Csm6 by cyclic tetra-adenylate. EMBO J. **43**, 304–315. (10.1038/s44318-023-00017-w) 38177499 PMC10897365

[27] Jia N, Jones R, Yang G, Ouerfelli O, Patel DJ. 2019 CRISPR-Cas III-A Csm6 CARF domain is a ring nuclease triggering stepwise cA4 cleavage with ApA>p formation terminating RNase activity. Mol. Cell **75**, 944–956. (10.1016/j.molcel.2019.06.014) 31326273 PMC6731128

[28] Binder SC, Schneberger N, Schmitz M, Engeser M, Geyer M, Rouillon C, Hagelueken G. 2024 The SAVED domain of the type III CRISPR protease CalpL is a ring nuclease. Nucleic Acids Res. **52**, 10520–10532. (10.1093/nar/gkae676) 39166476 PMC11417357

[29] Smalakyte D, Ruksenaite A, Sasnauskas G, Tamulaitiene G, Tamulaitis G. 2024 Filament formation activates protease and ring nuclease activities of CRISPR SAVED-Lon. Mol. Cell **84**, 4239–4255. (10.1016/j.molcel.2024.09.002) 39362215

[30] Cook R *et al*. 2021 INfrastructure for a PHAge REference database: identification of large-scale biases in the current collection of cultured phage genomes. PHAGE **2**, 214–223. (10.1089/phage.2021.0007) 36159887 PMC9041510

[31] Mölder F *et al*. 2021 Sustainable data analysis with Snakemake. F1000Research **10**, 33. (10.12688/f1000research.29032.1) 34035898 PMC8114187

[32] Russel J, Pinilla-Redondo R, Mayo-Muñoz D, Shah SA, Sørensen SJ. 2020 CRISPRCasTyper: automated identification, annotation, and classification of CRISPR-Cas loci. CRISPR J. **3**, 462–469. (10.1089/crispr.2020.0059) 33275853

[33] Eddy SR. 2011 Accelerated profile HMM searches. PLoS Comput. Biol. **7**, e1002195. (10.1371/journal.pcbi.1002195) 22039361 PMC3197634

[34] Edgar RC. 2022 Muscle5: high-accuracy alignment ensembles enable unbiased assessments of sequence homology and phylogeny. Nat. Commun. **13**, 6968. (10.1038/s41467-022-34630-w) 36379955 PMC9664440

[35] Price MN, Dehal PS, Arkin AP. 2010 FastTree 2 – approximately maximum-likelihood trees for large alignments. PLoS ONE **5**, e9490. (10.1371/journal.pone.0009490) 20224823 PMC2835736

[36] RStudio Team. 2020 RStudio: integrated development for R. RStudio (https://posit.co/download/rstudio-desktop/).

[37] Yu G. 2020 Using ggtree to visualize data on tree‐like structures. Curr. Protoc. Bioinform. **69**, e96. (10.1002/cpbi.96) 32162851

[38] . Rouillon C, Athukoralage JS, Graham S, Grüschow S, White MF. 2019 Investigation of the cyclic oligoadenylate signaling pathway of type III CRISPR systems. Methods Enzymol. 616, 191–218. (10.1016/bs.mie.2018.10.020) 30691643

[39] Abramson J *et al*. 2024 Accurate structure prediction of biomolecular interactions with AlphaFold 3. Nature **630**, 493–500. (10.1038/s41586-024-07487-w) 38718835 PMC11168924

[40] Holm L. 2022 Dali server: structural unification of protein families. Nucleic Acids Res. **50**, W210–W215. (10.1093/nar/gkac387) 35610055 PMC9252788

[41] Keller J *et al*. 2007 Crystal structure of AFV3-109, a highly conserved protein from crenarchaeal viruses. Virol. J. **4**, 12. (10.1186/1743-422X-4-12) 17241456 PMC1796864

[42] Du L, Zhang D, Luo Z, Lin Z. 2023 Molecular basis of stepwise cyclic tetra-adenylate cleavage by the type III CRISPR ring nuclease Crn1/Sso2081. Nucleic Acids Res. **51**, 2485–2495. (10.1093/nar/gkad101) 36807980 PMC10018336

[43] Laskowski RA, Jabłońska J, Pravda L, Vařeková RS, Thornton JM. 2018 PDBsum: structural summaries of PDB entries. Protein Sci. **27**, 129–134. (10.1002/pro.3289) 28875543 PMC5734310

[44] Huiting E *et al*. 2023 Bacteriophages inhibit and evade cGAS-like immune function in bacteria. Cell **186**, 864–876.(10.1016/j.cell.2022.12.041) 36750095 PMC9975087

[45] Jenson JM, Li T, Du F, Ea CK, Chen ZJ. 2023 Ubiquitin-like conjugation by bacterial cGAS enhances anti-phage defence. Nature **616**, 326–331. (10.1038/s41586-023-05862-7) 36848932 PMC10097602

[46] McMahon SA, Zhu W, Graham S, Rambo R, White MF, Gloster TM. 2020 Structure and mechanism of a type III CRISPR defence DNA nuclease activated by cyclic oligoadenylate. Nat. Commun. **11**, 500. (10.1038/s41467-019-14222-x) 31980625 PMC6981274

[47] Rostøl JT, Xie W, Kuryavyi V, Maguin P, Kao K, Froom R, Patel DJ, Marraffini LA. 2021 The Card1 nuclease provides defence during type III CRISPR immunity. Nature **590**, 624–629. (10.1038/s41586-021-03206-x) 33461211 PMC7906951

[48] Zhu W, McQuarrie S, Grüschow S, McMahon SA, Graham S, Gloster TM, White MF. 2021 The CRISPR ancillary effector Can2 is a dual-specificity nuclease potentiating type III CRISPR defence. Nucleic Acids Res. **49**, 2777–2789. (10.1093/nar/gkab073) 33590098 PMC7969007

[49] Mogila I, Tamulaitiene G, Keda K, Timinskas A, Ruksenaite A, Sasnauskas G, Venclovas Č, Siksnys V, Tamulaitis G. 2023 Ribosomal stalk-captured CARF-RelE ribonuclease inhibits translation following CRISPR signaling. Science **382**, 1036–1041. (10.1126/science.adj2107) 38033086

[50] Rouillon C *et al*. 2023 Antiviral signalling by a cyclic nucleotide activated CRISPR protease. Nature **614**, 168–174. (10.1038/s41586-022-05571-7) 36423657

[51] Baca CF, Yu Y, Rostøl JT, Majumder P, Patel DJ, Marraffini LA. 2024 The CRISPR effector Cam1 mediates membrane depolarization for phage defence. Nature **625**, 1–8. (10.1038/s41586-023-06902-y) PMC10808066 38200316

[52] Grüschow S, Adamson CS, White MF. 2021 Specificity and sensitivity of an RNA targeting type III CRISPR complex coupled with a NucC endonuclease effector. Nucleic Acids Res. **49**, 13122–13134. (10.1093/nar/gkab1190) 34871408 PMC8682760

[53] Grüschow S, McQuarrie S, Ackermann K, McMahon S, Bode BE, Gloster TM, White MF. 2024 CRISPR antiphage defence mediated by the cyclic nucleotide-binding membrane protein Csx23. Nucleic Acids Res. **52**, 2761–2775. (10.1093/nar/gkae167) 38471818 PMC11014256

[54] Millman A, Melamed S, Amitai G, Sorek R. 2020 Diversity and classification of cyclic-oligonucleotide-based anti-phage signalling systems. Nat. Microbiol. **5**, 1608–1615. (10.1038/s41564-020-0777-y) 32839535 PMC7610970

[55] Lau RK *et al*. 2020 Structure and mechanism of a cyclic trinucleotide-activated bacterial endonuclease mediating bacteriophage immunity. Mol. Cell **77**, 723–733. (10.1016/j.molcel.2019.12.010) 31932164 PMC7065454

[56] Hogrel G, Guild A, Graham S, Rickman H, Grüschow S, Bertrand Q, Spagnolo L, White MF. 2022 Cyclic nucleotide-induced helical structure activates a TIR immune effector. Nature **608**, 808–812. (10.1038/s41586-022-05070-9) 35948638

[57] Duncan-Lowey B, Kranzusch PJ. 2022 CBASS phage defense and evolution of antiviral nucleotide signaling. Curr. Opin. Immunol. **74**, 156–163. (10.1016/j.coi.2022.01.002) 35123147

[58] Cohen D, Melamed S, Millman A, Shulman G, Oppenheimer-Shaanan Y, Kacen A, Doron S, Amitai G, Sorek R. 2019 Cyclic GMP–AMP signalling protects bacteria against viral infection. Nature **574**, 691–695. (10.1038/s41586-019-1605-5) 31533127

[59] Krüger L, Gaskell-Mew L, Graham S, Shirran S, Hertel R, White MF. 2024 Reversible conjugation of a CBASS nucleotide cyclase regulates bacterial immune response to phage infection. Nat. Microbiol. **9**, 1–14. (10.1038/s41564-024-01670-5) 38589469 PMC11153139

[60] Tesson F, Hervé A, Mordret E, Touchon M, d’Humières C, Cury J, Bernheim A. 2022 Systematic and quantitative view of the antiviral arsenal of prokaryotes. Nat. Commun. **13**, 2561. (10.1038/s41467-022-30269-9) 35538097 PMC9090908

[61] Williams MC, Reker AE, Margolis SR, Liao J, Wiedmann M, Rojas ER, Meeske AJ. 2023 Restriction endonuclease cleavage of phage DNA enables resuscitation from Cas13-induced bacterial dormancy. Nat. Microbiol. **8**, 400–409. (10.1038/s41564-022-01318-2) 36782027 PMC9992242

[62] Hoikkala V. 2024 ring_nucleases. See https://github.com/vihoikka/ring_nucleases.

[63] Hoikkala V, Chi H, Grüschow S, Graham S, White M. 2025 Supplementary material from: Diversity and abundance of ring nucleases in type III CRISPR-Cas loci. Figshare. (10.6084/m9.figshare.c.7921347) PMC12409355 40904116

